# Rapid COVID-19 Screening Based on Self-Reported Symptoms: Psychometric Assessment and Validation of the EPICOVID19 Short Diagnostic Scale

**DOI:** 10.2196/23897

**Published:** 2021-01-06

**Authors:** Luca Bastiani, Loredana Fortunato, Stefania Pieroni, Fabrizio Bianchi, Fulvio Adorni, Federica Prinelli, Andrea Giacomelli, Gabriele Pagani, Stefania Maggi, Caterina Trevisan, Marianna Noale, Nithiya Jesuthasan, Aleksandra Sojic, Carla Pettenati, Massimo Andreoni, Raffaele Antonelli Incalzi, Massimo Galli, Sabrina Molinaro

**Affiliations:** 1 Institute of Clinical Physiology National Research Council Pisa Italy; 2 Institute of Biomedical Technologies National Research Council Milano Italy; 3 Infectious Diseases Unit Department of Biomedical and Clinical Sciences Luigi Sacco Università di Milano Milano Italy; 4 Institute of Neuroscience Aging Branch National Research Council Padova Italy; 5 Geriatric Unit Department of Medicine University of Padova Padova Italy; 6 Infectious Diseases Clinic Department of System Medicine Tor Vergata University of Rome Rome Italy; 7 Unit of Geriatrics Department of Medicine Biomedical Campus of Rome Rome Italy

**Keywords:** COVID-19, screening, diagnostic scale, validation, assessment, diagnostic, symptom, survey, algorithm

## Abstract

**Background:**

Confirmed COVID-19 cases have been registered in more than 200 countries, and as of July 28, 2020, over 16 million cases have been reported to the World Health Organization. This study was conducted during the epidemic peak of COVID-19 in Italy. The early identification of individuals with suspected COVID-19 is critical in immediately quarantining such individuals. Although surveys are widely used for identifying COVID-19 cases, outcomes, and associated risks, no validated epidemiological tool exists for surveying SARS-CoV-2 infection in the general population.

**Objective:**

We evaluated the capability of self-reported symptoms in discriminating COVID-19 to identify individuals who need to undergo instrumental measurements. We defined and validated a method for identifying a cutoff score.

**Methods:**

Our study is phase II of the EPICOVID19 Italian national survey, which launched in April 2020 and included a convenience sample of 201,121 adults who completed the EPICOVID19 questionnaire. The Phase II questionnaire, which focused on the results of nasopharyngeal swab (NPS) and serological tests, was mailed to all subjects who previously underwent NPS tests.

**Results:**

Of 2703 subjects who completed the Phase II questionnaire, 694 (25.7%) were NPS positive. Of the 472 subjects who underwent the immunoglobulin G (IgG) test and 421 who underwent the immunoglobulin M test, 22.9% (108/472) and 11.6% (49/421) tested positive, respectively. Compared to NPS-negative subjects, NPS-positive subjects had a higher incidence of fever (421/694, 60.7% vs 391/2009, 19.5%; *P*<.001), loss of taste and smell (365/694, 52.6% vs 239/2009, 11.9%; *P*<.001), and cough (352/694, 50.7% vs 580/2009, 28.9%; *P*<.001). With regard to subjects who underwent serological tests, IgG-positive subjects had a higher incidence of fever (65/108, 60.2% vs 43/364, 11.8%; *P*<.001) and pain in muscles/bones/joints (73/108, 67.6% vs 71/364, 19.5%; *P*<.001) than IgG-negative subjects. An analysis of self-reported COVID-19 symptom items revealed a 1-factor solution, the EPICOVID19 diagnostic scale. The following optimal scores were identified: 1.03 for respiratory problems, 1.07 for chest pain, 0.97 for loss of taste and smell 0.97, and 1.05 for tachycardia (ie, heart palpitations). These were the most important symptoms. For adults aged 18-84 years, the cutoff score was 2.56 (sensitivity: 76.56%; specificity: 68.24%) for NPS-positive subjects and 2.59 (sensitivity: 80.37%; specificity: 80.17%) for IgG-positive subjects. For subjects aged ≥60 years, the cutoff score was 1.28, and accuracy based on the presence of IgG antibodies improved (sensitivity: 88.00%; specificity: 89.58%).

**Conclusions:**

We developed a short diagnostic scale to detect subjects with symptoms that were potentially associated with COVID-19 from a wide population. Our results support the potential of self-reported symptoms in identifying individuals who require immediate clinical evaluations. Although these results come from the Italian pandemic period, this short diagnostic scale could be optimized and tested as a screening tool for future similar pandemics.

## Introduction

SARS-CoV-2 has led to a global pandemic; on July 28, 2020, over 16 million cases and 650,805 deaths across more than 200 countries were reported by the World Health Organization and Johns Hopkins Center for Health Security [1,2]. Italy was the first European country to be hit hard by the COVID-19 epidemic. It was also the European country with the highest number of COVID-19 deaths recorded (ie, 24,780 as of April 27, 2020) [3]. Besides the immediate human toll, the readily acknowledged and potentially long-lasting effects of the pandemic on global economies, politics, health, and privacy policies at many levels has extended beyond the development of vaccines and treatments. The rapid spread of the COVID-19 disease and its seemingly high degree of variability in its presentation among individuals has led to a level of clinical and scientific focus that has not been previously seen. This focus has encompassed both traditionally reviewed and preprint publications and resources. Collaborative groups are being formed at the local, regional, national, and international levels to address patient data collection, aggregation, and analysis in ways that may change the way research is carried out in the future [4]. To ensure that these efforts are both effective and productive, data must be evaluated in a way that is suitable for their inclusion in these activities, while still recognizing that what we understand about COVID-19 is much less than what we do not understand [5].

Due to the far-reaching scope of the pandemic, we are already confronting (1) the need to implement individual testing at a level far above current capacities to optimize individual treatment, assess disease spread, and anticipate potential strains on health care resources and personnel [6]; (2) the need for improvements in available tests, such as nasopharyngeal swab (NPS) and antibody detection tests, (ie, improvements in accuracy, specificity, and sensitivity) to enable the reliable evaluation and interpretation of data for use in clinical care and policy decisions [7]; and (3) the need to harmonize clinical observations and definitions to support the development of guidelines and prognostic and diagnostic indicators, and to develop a comprehensive understanding of COVID-19 and critical factors that can help differentiate between different patient susceptibilities, presentations of the disease, and responses to treatment [8,9].

The use of web-based surveys can greatly enhance access to broader populations in a cost-effective manner, optimize screening for individuals who may need immediate care, and provide an approach for achieving item 3 in the previous paragraph. A cross-sectional national survey, EPICOVID19, was launched on April 13, 2020 and received more than 200,000 responses [10]. The survey, which represents phase I of this study, was promoted through social media (ie, Facebook, Twitter, Instagram, and WhatsApp), press releases, internet pages, local radio and television stations, and institutional websites that called upon volunteers to contact the study website. The inclusion criteria were as follows: age of >18 years; access to a mobile phone, computer, or tablet with internet connectivity; and on-line consent to participate in this study.

This study was conducted during the epidemic peak of COVID-19 in Italy. The aim of our study was to assess the capability of the self-reported symptoms collected through the EPICOVID19 questionnaire in discriminating COVID-19 among symptomatic subjects, in order to identify individuals with suspected COVID-19 who need to undergo instrumental measurements and clinical examinations (ie, phase II of the EPICOVID19 study). The final objectives were proposing a method for the development of a total score for the self-reported symptoms in the EPICOVID19 questionnaire, and validating the scoring method based on molecular and serological clinical diagnosis data.

## Methods

### Study Design and Participants

**Figure 1:**
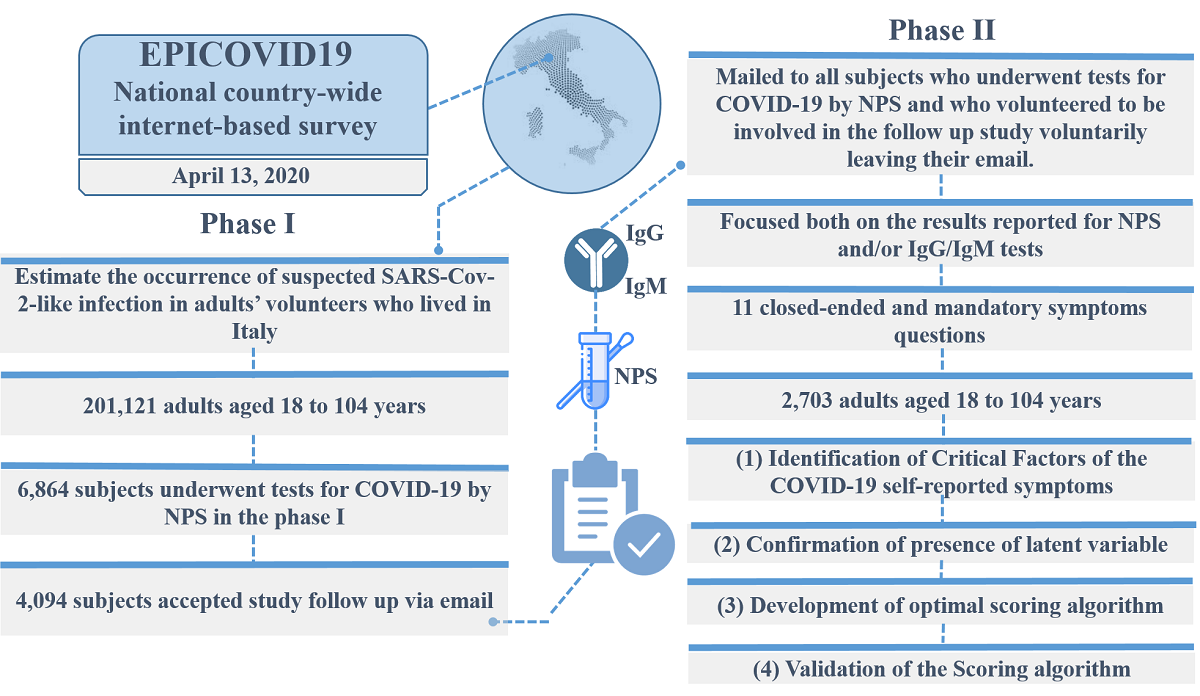
Overview of the EPICOVID19 2-phase study. IgG: immunoglobulin G; IgM: immunoglobulin M; NPS: nasopharyngeal swab.

Our study is phase II of the EPICOVID19 Italian national survey [9] (pages 1-8 in Multimedia Appendix 1), which launched in April 2020 and included a convenience sample of 201,121 adults who completed the EPICOVID19 questionnaire. Figure 1 shows the overview of the EPICOVID19 2-phase study. The Phase I questionnaire investigated 6 areas through 38 questions. The 6 areas were as follows: (1) sociodemographic characteristics, (2) clinical evaluation, (3) personal characteristics and health status, (4) housing conditions, (5) lifestyle, and (6) behaviors after the lockdown.

The Phase II questionnaire was mailed to all subjects who underwent NPS testing for COVID-19 and volunteered to be involved in the follow-up study in their phase I response. Phase II focused on the results of NPS and serological immunoglobulin G **(**IgG)/immunoglobulin M (IgM) tests and self-reported symptoms, with the aim of better identifying both symptomatic and asymptomatic SARS-CoV-2 infection cases [10].

Phase II was implemented by using an open-source statistical survey framework, LimeSurvey (version 3.17). This is a PHP (Hypertext Preprocessor)–based framework that is distributed under the GNU General Public License.

In phase II, responses to 11 questions were required. These questions covered the administration of the NPS and serological tests and the time that elapsed between observed/reported symptoms and clinical examination (ie, NPS and IgG/IgM tests) (pages 1-8 in Multimedia Appendix 1).

Of the 6864 subjects who underwent NPS testing for COVID-19 in phase I, 4094 subjects were invited by email to complete the Phase II questionnaires via the internet. Of these 4094 subjects, 38 could not participate because their email invitations were not delivered due to various issues (eg, wrong email address, full mailbox, host or domain name not found, etc), 101 refused to provide consent, and 1252 received the email, but did not proceed to complete the questionnaire.

The web-based survey included questions with close-ended answers in order to facilitate questionnaire compilation and avoid errors in digitizing answer values. At the end of the Italian lockdown period on May 2, 2020, the survey was closed and all collected data were exported for analysis with statistical tools. The base data for the statistical analysis was structured as a table that contained 1 row for each survey participant and as many columns as the collected responses. The questionnaire is available in pages 1-16 in Multimedia Appendix 1.

A total of 2703 subjects (response rate: 66%) completed the Phase II survey. After considering the 6864 subjects who underwent the NPS test in the Phase I survey, we compared the characteristics of 2703 respondents and 4161 nonrespondents. Respondents and nonrespondents to the Phase II survey appeared similar with respect to gender, age, the perception of their own health, and self-reported comorbidities. The details of the comparison between these 2 groups of subjects are included in page 9 in Multimedia Appendix 1. The resulting data of the 2703 subjects who completed the Phase II questionnaire were linked to the self-reported symptom results of the Phase I EPICOVID19 questionnaire, which included questions on the presence of 11 symptoms.

### Statistical Analysis

We analyzed the self-reported symptoms that were collected in the survey to define a method for calculating a total score and validate the scoring method for serological and molecular clinical diagnoses. This was done by using 4 standard questionnaire validation steps.

The first step was the identification of critical factors. We determined the factorial structure of the COVID-19 self-reported symptom items via exploratory factor analysis (EFA), followed by confirmatory factor analysis (CFA). EFA and parallel analysis were performed to evaluate the performance of specific symptoms (ie, loadings) and define the number of factors underlying these loadings.

The second step was the confirmation of the presence of latent variables. We carried out CFA via structural equation modelling to confirm the presence of 1 latent variable (ie, factor) underlying the 11 symptoms that were chosen to identify COVID-19. Several goodness-of-fit criteria were used, as follows: (1) standardized root mean square residual (SRSR); (2) root mean square error of approximation (RMSEA), which could not be >0.10; (3) comparative fit index (CFI); and (4) Tucker-Lewis index (TLI), which could not be <0.90.

The third step was the development of an optimal scoring algorithm. We developed an optimal scoring algorithm via homogeneity analysis by means of alternating least squares (HOMALS) and multiple correspondence analysis (MCA). Through the HOMALS procedure, we replaced specific dichotomous responses (ie, Yes/No) with categorical quantifications; the resulting score was the sum of the subject’s symptom responses after they were recoded based on category quantifications.

The fourth step was the validation of the scoring algorithm. We validated the score by using an external objective criterion that was based on receiver operating characteristics analysis, in order to evaluate the performance of COVID-19 symptom scores in distinguishing symptomatic individuals in the complete sample (ie, participants aged between 18 and 84 years) and 2 specific age groups (ie, participants aged <60 years and ≥60 years). Since we aimed to discriminate COVID-19 cases, we calculated the sensitivity, specificity, and Youden index with the following 2 reference standards: (1) subjects who tested positive in the NPS tests versus subjects who tested negative in the NPS test, and (2) subjects who tested positive in the serological IgG tests versus subjects who tested negative in the IgG test. The overall predictive performance was evaluated via area under the curve (AUC) analysis.

All statistical analyses were carried out using R software (version 3.6.3), IBM SPSS 23 (IBM Corp), and Stata Statistical Software (Release 15; StataCorp LLC). The details of the performed statistical analyses are reported in pages 10-12 in Multimedia Appendix 1.

### Ethical Approval

The Phase II EPICOVID19 study was approved by the Ethical Committee of the Istituto Nazionale per le Malattie Infettive, Institute for Research, Hospitalization and Healthcare Lazzaro Spallanzani as an amendment of the EPICOVID19 epidemiological study (approval number 93 in the trial register). Data transfer was safeguarded by means of password protection and encryption/decryption policies. All data were handled and stored in accordance with the European General Data Protection Regulation 2016/679 [11]. Informed consent details were accessible on the home page of the platform, and participants were asked to review these details before starting the Phase II questionnaire. The home page explained the purpose of the study, which data were to be collected, and how data were stored.

Subjects’ email addresses were the personal data provided on a voluntary basis in phase I. In our study, email addresses were only used to (1) send email invitations for participating in the Phase II survey and (2) link the information related to NPS and IgG/IgM test results to the information on symptoms collected during the phase I survey. In the participation mail, subjects were able to participate by clicking on the provided link to the survey, not participate by ignoring the invitation, communicate with the authors by using the provided study-specific email address, and request the deletion of their email address from the database.

## Results

### Study Design and Participants

The characteristics and NPS, IgG, and IgM results of the 2703 subjects, which were supplied by those who completed the Phase II survey, are shown in Table 1. The sample predominantly consisted of women (1841/2703, 68.1%), and the average age was 49 years (SD 15.0 years) and 52 years (SD 14.1 years) for women and men, respectively. Of the 2703 respondents, 151 (5.6%) had a low educational status, 837 (31%) had a medium educational status, and 1715 (63.4%) had a high educational status. The most reported chronic condition by participants was hypertension (361/2703, 13.4%), followed by immune system diseases (266/2703, 9.8%), and depression and anxiety diseases (194/2703, 7.2%). The least frequently reported chronic symptoms were liver (21/2703, 0.8%) and kidney (22/2703, 0.8%) diseases. All the details are reported in page 13 in Multimedia Appendix 1.

### Statistical Analysis

Of the 2703 subjects, 694 (25.7%) tested positive in the NPS test. Of these 694, 84 (12.1%) were asymptomatic. With regard to the subgroup of subjects who underwent serological testing, 472 underwent the IgG test and 421 underwent the IgM test, and 22.9% (108/472) and 11.6% (49/421) tested positive, respectively. Of the 108 IgG-positive subjects, 1 (0.9%) was asymptomatic. Of the 49 IgM-positive subjects, 5 (10.2%) were asymptomatic. With regard to NPS-positive subjects, the average number of days between initial symptoms and the day of swab execution was 9.3 days (SD 9.4 days; median 7 days, IQR 3-7 days). With regard to IgG-positive subjects, the average number of days between initial symptoms and the day of serological test execution was 36.1 days (SD 15.1 days; median 36.5 days, IQR 28-47 days). With regard to IgM-positive subjects, the average number of days from initial symptoms to the day of serological test execution was 26.1 days (SD 17.9 days; median 28 days, IQR 4-40 days). The incidence rate of the 11 symptoms reported by the 3 groups (ie, the NPS, IgG, IgM test groups) was similar between men and women. In the NPS-positive group, women only had a higher incidence of sore throat and cold and tachycardia (ie, heart palpitations) than men. In the IgG-positive group, men only had a higher incidence of headaches than women. In the IgM-positive group, women had a lower incidence of symptoms related to conjunctivitis than men.

The frequency of symptoms among NPS-positive subjects (Table 1) ranged from low rates of observation (eg, tachycardia [ie, heart palpitations]: 120/694, 17.3%; conjunctivitis: 111/694, 16%) to high rates of observation (eg, fever: 421/694, 60.7%; olfactory and taste disorders: 365/694, 52.6%). For all symptoms apart from headache, the incidence rates were significantly higher in NPS-positive subjects than in NPS-negative subjects (*P*<.001). With regard to the subgroup of individuals who underwent serological tests, the symptoms with a high incidence among subjects who tested positive were fever (IgG-positive group: 65/108, 60.2%; IgM-positive group: 28/49, 57.1%) and pain in muscles, bones, and joints (IgG-positive group: 73/108, 67.6%; IgM-positive group: 27/49, 55.1%). In the IgG serological test group, no significant difference was observed in the incidence of sore throat and cold symptoms (*P*=.23) between IgG-positive and IgG-negative subjects. The incidence of respiratory difficulty (*P*=.35), chest pain (*P*=.35), and gastrointestinal symptoms (*P*=.08) did not significantly differ between IgM-positive and IgM-negative subjects.

**Table 1:** Self-reported characteristics that were obtained from the Phase II survey and analyzed by using SARS-CoV-2 infection test results (N=2703).^a^

Variable		SARS-CoV-2 tests										
		Nasopharyngeal swab test, n=2703				Immunoglobulin G antibody test, n=472				Immunoglobulin M antibody test, n=421		
		Tested positive	Tested negative	*P* value	Tested positive		Tested negative	*P* value	Tested positive		Tested negative	*P* value
Number, n (%)		694 (25.7)	2009 (74.3)	N/A^b^	108 (22.9)		364 (77.1)	N/A	49 (11.6)		372 (88.4)	N/A
Women, n (%)		440 (63.4)	1401 (69.7)	.001	61 (56.5)		258 (70.9)	.005	25 (51)		260 (69.9)	.008
Age (years), mean (SD)		55.5 (18.06)	47.55 (12.81)	<.001	48.8 (11.74)		45.5 (11.49)	.009	50.6 (10.56)		45.8 (11.69)	.008
**Answered questions on symptoms, n (%)**												
	Fever with a temperature of >37.5°C for at least 3 consecutive days	421 (60.7)	391 (19.5)	<.001	65 (60.2)		43 (11.8)	<.001	28 (57.1)		68 (18.3)	<.001
	Cough	352 (50.7)	580 (28.9)	<.001	63 (58.3)		76 (20.9)	<.001	26 (53.1)		95 (25.5)	<.001
	Sore throat and cold	232 (33.4)	756 (37.6)	.048	46 (42.6)		132 (36.3)	.233	16 (32.7)		135 (36.3)	.62
	Headache	313 (45.1)	703 (35)	<.001	61 (56.5)		96 (26.4)	<.001	23 (46.9)		117 (31.5)	.03
	Pain in muscles, bones, and joints	360 (51.9)	572 (28.5)	<.001	73 (67.6)		71 (19.5)	<.001	27 (55.1)		98 (26.3)	<.001
	Loss of taste and smell	365 (52.6)	239 (11.9)	<.001	66 (61.1)		29 (8)	<.001	21 (42.9)		55 (14.8)	<.001
	Respiratory difficulty (ie, sense of breathlessness at rest)	179 (25.8)	249 (12.4)	<.001	21 (19.4)		28 (7.7)	<.001	7 (14.3)		37 (9.9)	.35
	Chest pain (ie, sternum pain)	136 (19.6)	251 (12.5)	<.001	26 (24.1)		25 (6.9)	<.001	7 (14.3)		37 (9.9)	.35
	Tachycardia (ie, heart palpitations)	120 (17.3)	237 (11.8)	<.001	24 (22.2)		27 (7.4)	<.001	10 (20.4)		31 (8.3)	.007
	Gastrointestinal complaints (ie, diarrhea, nausea, and vomiting)	289 (41.6)	452 (22.5)	<.001	54 (50)		65 (17.9)	<.001	17 (34.7)		87 (23.4)	.08
	Conjunctivitis (ie, red eyes)	111 (16)	221 (11)	<.001	24 (22.2)		35 (9.6)	.001	11 (22.4)		40 (10.8)	.02

^a^Mean (SD) was used for continuous variables, which were analyzed with an independent 2-tailed *t* test, and n (%) was used for categorical variables, which were analyzed with a Chi-square test.

^b^N/A: not applicable.

The EFA, which involved the principal-component factors and Horn parallel analysis methods, pointed out 1 factor. Eigenvalues, descriptive indices, and goodness-of-fit indices for the cumulative percentage of explained data variability obtained through EFA are displayed in Table 2. Principal-component factors analysis only highlighted 1 factor with an 89.9% proportion of explained variability, while the Horn parallel analysis identified 2 factors with eigenvalues of >1.0 and a 49.8% and 10.3% proportion of explained variability, respectively.

**Table 2:** Descriptive and goodness-of-fit dimensionality indices from the exploratory factor analysis of the 11 EPICOVID19 symptoms reported by 2703 subjects, based on the principal-component factors and Horn parallel analysis methods with an eigenvalue of >1.

Factor	Exploratory factor analysis					
	Principal-component factors analysis			Horn parallel analysis		
	Eigenvalue	Proportion of explained variability	Cumulative explained variability	Eigenvalue	Proportion of explained variability	Cumulative explained variability
1	5.00	89.9%	89.9%	5.48	49.8	49.8%
2	N/A^a^	N/A	N/A	1.14	10.3	60.1%

^a^N/A: not applicable.

**Figure 2:**
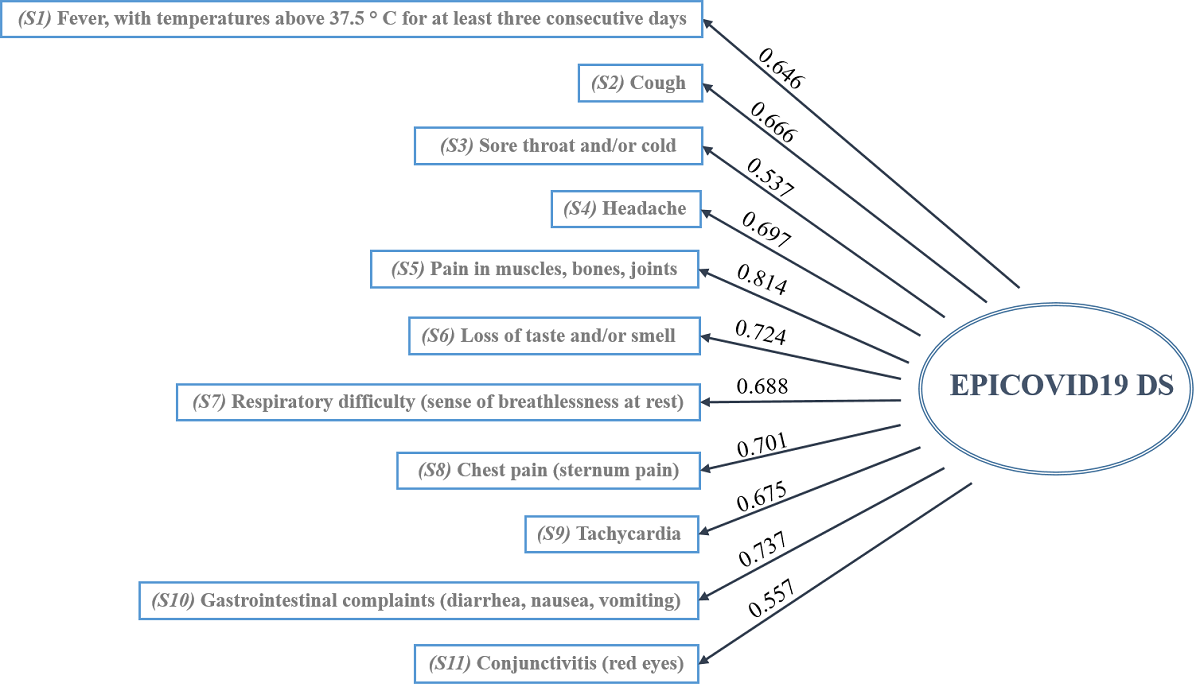
Standardized factor loading values of the 1-factor model, EPICVOID19 DS. The goodness-of-fit indices are as follows: a standardized root mean square residual of 0.072, root mean square error of approximation of 0.052, comparative fit index of 0.977, and Tucker-Lewis index of 0.971. EPICOVID19 DS: EPICOVID19 diagnostic scale.

Based on a priori determined cutoff value, a factor loading of >0.35 was maintained. The factor loading rule of the 1-factor solution extracted from the principal-component factors analysis is available in page 13 in Multimedia Appendix 1. The dimensionality indices of the 1-factor solution, which had a high cumulative and proportion of explained variability (89.9%), confirmed the presence of 1 latent variable underlying COVID-19 symptom items. Therefore, we defined the 1-factor solution as the EPICOVID19 diagnostic scale (EPICOVID19 DS). Based on our CFA results, we confirmed that the latent construct was unidimensional and determined how the variables contributed to the EPICOVID19 DS. Figure 2 shows the values of the standardized factor loadings for the 1-factor model. The magnitude of each factor loading value was >0.4, which indicated the importance of the corresponding item to the EPICOVID19 DS. For example, pain in muscles, bones, and joints was the most important variable, with a factor loading value of 0.814. The other variables with an optimal specific validity index were respiratory difficulty (sense of breathlessness at rest: 0.688; loss of taste and smell: 0.724) and gastrointestinal complaints, with item-factor correlations of 0.737. The lowest values were observed for the sore throat and cold and conjunctivitis items, which had a specific validity index of 0.537 and 0.557, respectively. The goodness of fit (ie, SMSR and RMSEA) of the EPICOVID19 DS was acceptable, because 2 indices were <0.10 (SMSR 0.072; RMSEA 0.052; CFI 0.977; TLI 0.971). We computed CFA indices to measure the internal validity of the model (page 14 in Multimedia Appendix 1).

Given the successful unidimensionality testing of the EPICOVID19 DS, optimal scaling was performed. The proposed optimal score was extracted from the HOMALS procedure (ie, single-factor measurement), and for each subject, the computed optimal score was obtained by summing the category quantifications of the screening questionnaire item responses. Cronbach (α=0.88) and Greenacre (statistic=78%) indices confirmed the unidimensionality found in the EFA and CFA. The HOMALS optimal category quantifications of the EPICOVID19 symptom variables are summarized in Table 3, which has columns for the binary options (ie, Yes/No) and rows for the different symptoms. The HOMALS category quantifications were scaled so that the score obtained from the sum of responses would range from 0 (ie, if a subject answered “No” to all the symptoms) to 10 (ie, if a subject answered “Yes” to all the symptoms). These values are shown in the last column of Table 3. An example of a resulting score calculation is as follows: if the subject response pattern with respect to symptoms is “Yes, No, Yes, No, No, Yes, Yes, No, No, No, Yes,” the corresponding recoded response pattern is 0.80, 0, 0.64, 0, 0, 0.97, 1.03, 0, 0, 0, 0.88, and the subject’s optimal score would be calculated as 0.8 + 0 + 0.64 + 0 + 0 + 0.97 + 1.03 + 0 + 0 + 0 + 0.88 = 4.2.

**Table 3:** Multiple correspondence analysis optimal weights for the recoding of the EPICOVID19 diagnostic scale.

Symptoms	HOMALS^a^ category quantifications		Recoded HOMALS category quantifications	
	No	Yes	No	Yes
Fever with a temperature of >37.5°C for at least 3 consecutive days	−0.362	0.8421	0	0.80
Cough	−0.426	0.810	0	0.81
Sore throat and/or cold	−0.358	0.622	0	0.64
Headache	−0.470	0.780	0	0.83
Pain in muscles, bones, and joints	−0.505	0.959	0	0.97
Loss of taste and/or smell	−0.326	1.133	0	0.97
Respiratory difficulty (ie, sense of breathlessness at rest)	−0.246	1.305	0	1.03
Chest pain (ie, sternum pain)	−0.232	1.388	0	1.07
Tachycardia (ie, heart palpitations)	−0.209	1.374	0	1.05
Gastrointestinal complaints (ie, diarrhea, nausea, and vomiting)	−0.393	1.042	0	0.95
Conjunctivitis (ie, red eyes)	−0.164	1.170	0	0.88

^a^HOMALS: homogeneity analysis by means of alternating least squares.

There was no significant difference in the mean EPICOVID19 DS score between men (mean 2.34, SD 2.2) and women (mean 2.49, SD 2.4) (*P*=.14). A low negative correlation between the scores and ages of the participants was found (ρ=−0.126; *P*<.001). Of the 2703 subjects, 1738 (64.3%) reported no preexisting diseases, 684 (25.3%) only had 1 chronic condition, while the remaining 281 (10.4%) declared ≥2 conditions. Significant differences in the mean EPICOVID19 DS score were observed between participants who did not report any disease (mean 2.26, SD 2.3) and those with at least 1 preexisting condition (mean 2.75, SD 2.4) (*P*<.001). Based on our analysis of the mean EPICOVID19 DS score among healthy subjects and subjects with 1 chronic condition, we observed significant differences between healthy subjects and subjects with lung diseases (healthy subjects: mean 2.40, SD 2.3; subjects with lung diseases: mean 3.10, SD 2.5; *P*<.001), healthy subjects and subjects with immune system diseases (healthy subjects: mean 2.39, SD 2.3; subjects with immune system diseases: mean 2.91, SD 2.4; *P*<.001), and healthy subjects and subjects with depression and anxiety diseases (healthy subjects: mean 2.42, SD 2.4; subjects with depression and anxiety: mean 2.79, SD 2.6; *P*=.036). For the other chronic conditions (ie, heart disease: *P*=.22; hypertension: *P*=.59; kidney disease: *P*=.45; tumor: *P*=.13; metabolic disease: *P*=.52; liver disease: *P*=.64), no significant differences in mean EPICOVID19 DS score were found.

The screening properties of the EPICOVID19 DS were compared to those of COVID-19–positive molecular and serological tests. These are shown in Table 4. The best Youden index value was observed for EPICOVID19 DS, with respect to subjects diagnosed with COVID-19 via NPS testing. A good trade-off between sensitivity and specificity was observed (sensitivity: 76.56%; specificity: 68.24%; AUC 77.5, 95% CI 75.6-79.4). The cutoff score obtained was 2.56. The sensitivity and specificity of the EPICOVID19 DS improved when compared to those of COVID-19–positive IgG antibody test (sensitivity: 80.37%; specificity: 80.17%; AUC 86.0, 95% CI 82.3-89.5). The cutoff value obtained (2.59) was similar to that of the NPS-positive test. The positive and negative predictive values for the IgG-positive serological test (positive predictive value [PPV]: 54.43%; negative predictive value [NPV]: 93.27%) were higher than those of the NPS test (PPV: 42.26%; NPV: 90.55%). We observed a poor performance with regard to IgM test results, so these are not presented in Table 4.

**Table 4:** Sensitivity and specificity of the EPICOVID19 diagnostic scale compared to those of positive COVID-19 molecular and serological diagnoses (ie, for subjects aged 18-84 years).

Statistic	SARS-CoV-2 tests	
	Nasopharyngeal swab test (n=2703), value (95% CI)^a,b^	Immunoglobulin G antibody test (n=472), value (95% CI)^c,d^
Sensitivity, %	76.56 (72.99-79.87)	80.37 (71.58-87.42)
Specificity, %	68.24 (66.16-70.28)	80.17 (75.69-84.14)
Positive likelihood ratio	2.41 (2.23-2.61)	4.05 (3.23-5.08)
Negative likelihood ratio	0.34 (0.30-0.40)	0.24 (0.17-0.36)
COVID-19–positive tests, %	23.29 (21.68-24.96)	22.77 (19.05-26.83)
Positive predictive value, %	42.26 (40.38-44.17)	54.43 (48.77-59.98)
Negative predictive value, %	90.55 (89.23-91.74)	93.27 (90.40-95.33)
Accuracy, %	70.18 (68.39-71.93)	80.21 (76.32-83.72)

^a^There were 694 NPS-positive subjects.

^b^The cutoff value for the nasopharyngeal swab test was 2.59.

^c^There were 108 immunoglobulin G-positive patients.

^d^The cutoff value for the immunoglobulin G antibody test was 2.56.

When the EPICOVID19 DS scoring algorithm was applied to specific age groups, the sensitivity and specificity of the IgG-positive antibody test (sensitivity: 88.00%; specificity: 89.58%; AUC 93.10, 95% CI 86.0-99.5) improved greatly among subjects aged ≥60 years, and the obtained cutoff value (1.28) was lower than the cutoff value for the subjects aged <60 years (2.71; sensitivity: 88.00%; specificity: 89.58%; AUC 93.10, 95% CI 86.0-99.5). The PPV and NPV of the IgG test were higher for subjects aged ≥60 years (PPV: 81.48%; NPV: 93.48%) than those for subjects aged <60 years (PPV: 51.52%; NPV: 94.38%). Furthermore, we observed the same performance in the NPS test between the specific age groups (ie, aged ≥60 years and aged <60 years), with respect to the overall sample (ie, aged 18-84 years). The details of the screening properties of the EPICOVID19 DS compared to those of COVID-19–positive molecular and serological tests for specific age groups are reported in page 16 in Multimedia Appendix 1.

## Discussion

Our focus was on developing a tool composed of simple questions related to COVID-19 symptomatology for the identification of subjects who are more likely to be infected with SARS-CoV-2 in the general population. We validated the EPICOVID19 DS with a sample of voluntary subjects based on serological and molecular clinical diagnoses. The optimal score, which was computed for 2703 adults aged 18-84 years, discriminated symptomatic individuals. Before calculating the score, we performed both exploratory and confirmatory factor analyses to determine the number of factors/dimensions underlying the questionnaire. The results of these analyses supported the 1-factor model and the unidimensionality of the EPICOVID19 questionnaire. The magnitude of all factor loading values was satisfactory, and the highest factor loading values were observed for respiratory difficulty, chest pain, tachycardia (ie, heart palpitations), and loss of taste and smell. Furthermore, gastrointestinal complaint items appeared to be the most essential features of the EPICOVID19 DS. The high value for chest pain can also be explained by the fact that several patients reported it, possibly because of tracheal pain caused by pneumonia [12,13]. Several clinical studies on hospitalized patients have shown that, at the onset of COVID-19, patients frequently show typical symptoms of viral pneumonia [3]. Symptoms that are less common, but still reported by a substantial number of patients, are nasal congestion, sore throat, gastrointestinal complaints, and olfactory and taste disorders [14-16]. Subjects have often reported gastrointestinal complaints as concurrent symptoms instead of isolated symptoms of SARS-CoV-2 infection [17]*.* The lowest factor loading values were observed for sore throat and cold and conjunctivitis. These lower values may be related to the fact that conjunctivitis and cold are not the most frequent symptoms of COVID-19 [18]. In line with other recent studies [19,20], the features we encountered in this study showed various aspects of the definition for COVID-19 diagnosis. Cough, loss of taste and smell, and respiratory difficulty are among the most reported symptoms in previous studies, and they corresponded to the items that were the most important to our score [12,16,21,22].

The clinical presentation of COVID-19 varies, and discrepancies may exist between symptoms and the disease. A recent meta-analysis of the symptoms of 50,000 patients with COVID-19 found that fever and cough were the most common symptoms (incidence: 89.1% and 72.2%, respectively) [23], and a separate study on hospitalized subjects has suggested that respiratory distress has been reported in the most critical cases of COVID-19 [24]. With the aim of supporting medical decision making, predicted models have been developed for detecting people in the general population who are at risk of being admitted to hospital and diagnosing COVID-19 in patients with related symptoms. However, the results presented in a recent systematic review on such models describe poor research performance and a high risk of bias [25].

Based on our HOMALS, we proposed a scoring methodology for developing an improved scale. Therefore, we provided a numerical weight value (ie, optimal quantification) that represents the importance of the binary response categories (ie, Yes/No) for each question in the EPICOVID19 DS. As a result, the various binary items of the 11 questions in the EPICOVID19 DS contributed to the overall score, albeit with different weights. This produced an improved scale (ie, 0-10) that reflects the importance of each symptom. Thus, respiratory problems and chest pain were the most important symptoms, with a score of 1.03 and 1.07, respectively. The other symptoms that had an important contribution to the total score were gastrointestinal complaints (0.95), loss of taste and smell (0.97), and tachycardia (ie, heart palpitations) (1.05). Subsequently, we computed the sensitivity and specificity of EPICOVID19 DS compared to those of COVID-19–positive serological and molecular tests. For NPS-positive subjects, the cutoff score was 2.56, with a sensitivity of 76.56% and specificity of 68.24%. For IgG-positive subjects, the cutoff score was 2.59, and sensitivity, specificity, PPV, and NPV with respect to NPS-positive tests substantially improved (sensitivity: 80.37%; specificity: 80.17%; PPV: 54.43%; NPV: 93.27%). When the EPICOVID19 DS scoring algorithm was tested on subjects aged ≥60 years, the accuracy of IgG-positive antibody tests improved (sensitivity 88.00%; specificity 89.58%; AUC 93.10, 95% CI 86.0-99.5; PPV: 81.48%; NPV IgG 93.48%), and the threshold of detection (1.28) was lower than that of subjects aged <60 years.

Our data are consistent with the findings reported in previous studies. In mid-May 2020, the European all-cause mortality monitoring system showed that all-cause mortality was above the expected rate in several European countries (ie, Belgium, France, Malta, and Spain), including Italy [26], mainly for people aged ≥60 years. People aged ≥60 years are more vulnerable to SARS-CoV-2 infection, and those with preexisting medical conditions are particularly at risk. Several best practices for older people and their families have been recommended by the World Health Organization, Centers for Disease Control and Prevention, geriatricians, and infectious diseases specialists [27]. The sensitivity and specificity of serological and molecular diagnostic tests for COVID-19 have not been fully elucidated, but several studies have suggested that sensitivity could be as low as 80% [28,29]. This raises concerns of high false-negative rates, which could result in an increase in infection spread among the community. There is no absolute answer for the sensitivity and specificity of COVID-19 diagnostic tests, because to determine their accuracy, they must be compared with a gold-standard test, which does not currently exist. By considering estimates for sensitivity and specificity, PPVs and NPVs can be calculated based on disease prevalence and the rate of illness in the population. However, there is considerable uncertainty with regard to the prevalence of COVID-19 [30]. Statistically, it has been assumed that PPVs vary widely and range between 30-50% in areas with a low COVID-19 prevalence, as stated in a recent US study on COVID-19 [31].

Early recognition screening and rapid diagnosis are essential for preventing transmission and providing supportive care in a timely manner. Nevertheless, screening is different from further, more detailed diagnostic test assessments. This is of particular relevance, as resources for full testing remain limited, and optimizing the use of such resources is critical. The EPICOVID19 DS can be used as a preliminary assessment that attempts to detect subjects with symptoms that are potentially associated with COVID-19 among a wide population. The EPICOVID19 DS does not enable clinical interviews for determining complete symptomatic profiles and needs, but it does identify those who may warrant further assessment. Therefore, it would be advantageous to use the EPICOVID19 DS for screening in primary care settings, so that general practitioners can avoid people with suspected COVID-19 in primary care offices whenever possible [32]. The EPICOVID19 DS can also be used as an initial screening tool before patients are managed remotely via telephone or video consultations [33]. Additionally, the EPICOVID19 DS can be applied to the general population. Once a score is assigned to each symptom, the EPICOVID19 DS can allow for different cutoff values to be set, based on the subjects involved and the gold standards used (ie, NPS tests, serological tests, clinical evaluation by clinicians, etc).

It should be noted that since it is plausible to expect a lower prevalence rate in the general population than the 22.77% in this study, the probability of NPVs would increase beyond the current 93.27%. Consequently, the probability of progressing to COVID-19 for subjects who test negative (ie, 1 − NPV) would be less than the current 6.7%. Furthermore, although the identified symptoms in this study are not specific to COVID-19, they have been reported as valid references for a population setting, because they are frequently reported by patients with COVID-19. In a nonpandemic scenario, it is likely that these symptoms could be assessed with different weights because of their aspecificity, which would configure the EPICOVID19 DS as a valid diagnostic support tool for pandemic situations. Moreover, health authorities are still unable to use classic tests to monitor the spread of SARS-CoV-2 infection, and allowing the circulation of unsuspecting individuals with COVID-19 could represent a risk for the spread of the infection. The validation of an instrument that can easily identify a suspected COVID-19 case by attributing a score to each symptom related to COVID-19 can be of great importance in facilitating the containment of the epidemic. Our proposed cutoff score seems worthy of validation for use in broader populations to confirm its clinimetric properties. In the event of its validation, our cutoff score might be useful in selecting people who require serological and molecular diagnostic tests for COVID-19.

The availability and accessibility of diagnostic tests for the SARS-CoV-2 coronavirus have proven to be key in containing the COVID-19 pandemic. The early identification of subjects who test positive for COVID-19 (ie, via molecular and serological tests) among people with specific symptoms or people who are at risk is crucial for limiting the spread of the infection. The tool we validated responds to the need for readily identifying a suspected COVID-19 case, by attributing a score to each symptom related to COVID-19. Although our validation was satisfactory, our proposed cutoff score seems worthy of further testing in larger populations in order to confirm its clinimetric properties and usefulness in selecting people who require serological and molecular diagnostic tests for COVID-19.

Although the EPICOVID19 DS tool can be used as a public health prevention instrument, directing subjects to a self-assessment tool without warning may trigger panic, alarm, and concern among the screened population. Furthermore, the limitations of our study must be considered. First, participation in this study was voluntary, and the sample was not representative of the general population. This means that potential selection biases must be taken into consideration. Second, data were collected from a highly educated, young-adult convenience population sample with low multimorbidity. This was a result of the phase I EPICOVID19 study [10], and such a sample is expected in studies that involve a web-based questionnaire that is promoted via email invitation. Third, in the context of a pandemic, our survey might have interested people who had no opportunity to report symptoms to clinicians. Moreover, the effect of recall bias cannot be excluded among the participants who tested positive for COVID-19 or presented with symptoms related to SARS-COV2 infection. The fourth limitation of our study is the small sample size in the analysis of the 2 age groups (ie, subjects aged <60 and ≥60 years). Given these limitations, the adoption of the EPICOVID19 DS should be considered with caution. The procedures outlined for the development of the EPICOVID19 DS can be applied iteratively as new data is collected, to continue the refinement of this potentially valuable clinical decision support tool.

In conclusion, the proposed EPICOVID19 DS seems worthy of further testing in different scenarios and populations to achieve a comprehensive understanding of its clinimetric properties for both low-prevalence and high-prevalence COVID-19 settings, and its aptitude for capturing disease severity data. This will allow us to define the boundaries of its use and identify optimal indicators to assist clinicians with the early recognition of COVID-19.

## Appendix

Multimedia Appendix 1 Phase I EPICOVID19 questionnaire, Phase 2 Valid Symptoms Section of the EPICOVID19 questionnaire, and statistical appendix.

## Abbreviations

AUC: area under the curve
CFA: confirmatory factor analysis
CFI: comparative fit index
EFA: exploratory factor analysis
EPICOVID19 DS: EPICOVID19 diagnostic scale
HOMALS: homogeneity analysis by means of alternating least squares
IgG: immunoglobulin G
IgM: immunoglobulin M
NPS: nasopharyngeal swab
NPV: negative predictive value
PHP: hypertext preprocessor
PPV: positive predictive value
RMSEA: root mean square error of approximation
SRSR: standardized root mean square residual
TLI: Tucker-Lewis index
