# Supplementary material for: Rapid COVID-19 Screening Based on Self-Reported Symptoms: Psychometric Assessment and Validation of the EPICOVID19 Short Diagnostic Scale
Source: J Med Internet Res. 2021 Jan 6;23(1):e23897. doi: 10.2196/23897 (PMC7790735; doi:10.2196/23897)
Supplement: Multimedia Appendix 1 [file jmir_v23i1e23897_app1.docx]

**Supplementary appendix**

**This appendix formed part of the original submission**

**Phase I Epicovid-19 questionnaire and Phase 2 Valid Symptoms Section of Epicovid-19 questionnaire**

**COVID-19 NATIONAL EPIDEMIOLOGICAL SURVEY (EPICOVID19)**

**Phase I questionnaire**

**SOCIO-DEMOGRAPHIC DATA**

**mandatory questions*

1. **Sex at birth*** M |__| F |__|
2. **Year of birth *** |__|__|__|__|
3. **Ethnicity***

|__| Europe

|__| Asia

|__| North America

|__| Central/South America

|__| Africa

|__| Oceania

|__| Other

1. **Postal code*** |__|__|__|__|__|
2. **Municipality***  |__|__|__|__|__|__|__|__|__|__|__|__|__|__|__|__|__|__|__|__|__|__|__|__|__|
3. **Education***

|__| None

|__| Primary school

|__| Middle high school

|__| High school

|__| University degree

|__| Post graduate degree (PhD, vocational master, medical specialization)

1. **Occupational status***

|__| Employed

|__| Student

|__| Unemployed

|__| Retired

|__| Other

1. **Last occupation***

|__| Armed forces occupations

|__| Managers

|__| Intellectual and scientific professionals (e.g. clinicians, engineers, researchers, teachers)

|__| [Technicians](https://en.wikipedia.org/wiki/Technician) and associate professionals (e.g. healthcare technicians, IT technicians)

|__| Clerk

|__| Service and sales workers (e.g. merchants, sales persons)

|__| Craft and related trades workers

|__| Plant and machine operators, assemblers, and drivers of vehicles

|__| Other

**CLINICAL EVALUATION**

1. **Did you have one or more of the following symptoms since the 1st of February 2020? ***

|__| Fever with a temperature greater than 37.5 °C for at least three consecutive days

|__| Cough

|__| Sore throat/rhinorrea

|__| Headache

|__| Myalgia

|__| Olfactory or taste disorders

|__| Shortness of breath

|__| Chest pain

|__| Feelings of having a fast-beating

|__| Gastrointestinal disorders (diarrhoea, nausea, vomiting)

|__| Conjunctivitis

|__| Pneumoniae

1. **If you had at least one of the symptoms above, please indicate in which month they occurred for the first time ***

|__| February

|__| March

|__| April

1. **Have you ever been diagnosed with one or more of the following conditions?**

|__| Lung diseases (e.g. asthma, obstructive pulmonary disease)

|__| Heart diseases (e.g. ischemic heart disease, atrial fibrillation)

|__| Hypertension

|__| Kidney diseases

|__| Immune system diseases (e.g. thyroid disease, psoriasis, rheumatoid arthritis)

|__| Tumours

|__| Metabolic diseases (e.g. diabetes, obesity, gout)

|__| Liver diseases (e.g. hepatitis, cirrhosis, liver failure)

|__| Depression and/or anxiety

1. **Please indicate other conditions**

|__| Surgical procedures under general anaesthesia during the last year

|__| Transplants

|__| Allergies

|__| Pregnancy

|__| Non self-sufficient in carrying out daily activities

|__| Healthcare workers (e.g. clinicians, nurses, rescuer, pharmacist)

1. **Did you carry out the following vaccinations?***

- Flu shot during the last autumn |__| Yes |__| No

- Anti-pneumococcal in the last 12 months |__| Yes |__| No

- Other vaccinations in the last 12 months |__| Yes |__| No

1. **Do you regularly take one or more of the following medicines?**

|__| Aspirin

|__| Anti-hypertensive

|__| Hypocholesterolemic drugs

|__| Anti-diabetics

|__| Anti-cancer drugs

|__| Corticosteroids

|__| Thyroid drugs (e.g. euthyrox)

|__| Anti-inflammatory drugs

|__| Anxiety medications and/or sedatives

|__| Anti-depressant

|__| Supplements (e.g. vitamins)

*If females*

1. **Have you ever been taking birth pills and/or hormone replacement therapy?***

|__| No

|__| Yes, in the past, for less than 5 years

|__| Yes, in the past, for more than 5 years

|__| Yes currently, taking it less than 5 years

|__| Yes currently, taking it more than 5 years

1. **Indicate the number of completed pregnancies***

|__| 0

|__| 1

|__| 2

|__| 3 or more

1. **Have you been in a close contact (direct contact at a distance of less than 2 meters, or in a closed environment such as a house, workplace, transportation vehicles) with confirmed COVID-19 cases, live or deceased?***

|__| Yes |__| No

1. **Have you been in a close contact (direct contact at a distance of less than 2 meters, or in a closed environment such as a house, workplace, transportation vehicles) with suspected COVID-19 cases, live or deceased?***

|__| Yes |__| No |__| I don’t know

1. **Did you contact the emergency number and/or the general practitioner to report any symptoms of suspected infection by COVID-19?***

|__| No

|__| No but I went to the hospital on my own initiative

|__| Yes, and they suggested to me isolation

|__| Yes, and they did not suggest to me isolation

|__| Yes, and I was sent to the hospital

1. **Have you been tested for COVID-19? ***

|__| Yes, with a positive result

|__| Yes, with a negative result

|__| Yes, but I do not know the result

|__| No, I did not perform any test

1. **Have you been hospitalized due to COVID-19, either as a suspected or as a confirmed case?***

|__| Yes |__| No

1. **Please indicate other elements that might be of relevance for COVID-19** __________________________________________________________________________________________________________________________________________________________________________

**PERSONAL CHARACTERISTICS AND HEALTH STATUS**

1. **How would you describe your health in general?***

|__| Very bad |__| Bad |__| Adequate |__| Good |__| Very good

1. **Do you fear getting infected with the coronavirus (COVID-19)?***

|__| No

|__| Just a little bit

|__| Neutral

|__| Quite enough

|__| Yes, a lot

1. **Do you fear your family being infected with the coronavirus (COVID-19)?***

|__| No

|__| Just a little bit

|__| Neutral

|__| Quite enough

|__| Yes, a lot

**HOUSING CONDITIONS**

1. **Your home is located in**:*

|__| City centre with more than 100.000 inhabitants

|__| Suburbs of cities with more than 100.000 inhabitants

|__| Small town

|__| Countryside

1. **Your home is located in an area where the road traffic is**:*

|__| Intense (living near a busy road)

|__| Moderate

|__| Low

1. **How many rooms there are in your home (excluding the bathroom and auxiliary spaces)?***

|__| One |__| Two |__| Three |__| More than three

1. **Besides you, how many people live in your household?***

|__| None |__| One |__| Two |__| More than two

1. **Are there in the same household elderly persons or anyone with immunocompromising or chronic disease conditions?***

|__| Yes |__| No

**LIFESTYLE**

1. **How many people have you been in contact with in average, prior to the Governmental restrictions on lockdown?***

|__| Less than 10 |__| Between 10 and 100 |__| More than 100

1. **Do you smoke?***

|__| I have never smoked or I smoked less than 100 cigarettes in my lifetime

|__| I am a former smoker (I have smoked at least 100 cigarettes in my lifetime and I do not smoke anymore)

|__| Yes, smoking less than 10 cigarettes per day

|__| Yes, smoking between 10 and 20 cigarettes per day

|__| Yes, smoking more than 20 cigarettes per day (more than one pack per day)

*If former smoker*

1. **How many year have you smoked?*** |__|__|

*If current smoker*

1. **How many years?*** |__|__|
2. **Prior to the lockdown by the Governmental restrictions, for how long did you follow a regular routine of moderate or intense physical activity (swimming, racing)?***

|__| Not doing any physical activity or doing it for less than 10 minutes per week

|__| Between 10 minutes and two hours and half per week

|__| More than two hours and half per week

**BEHAVIOURS FOLLOWING THE LOCKDOWN**

1. **Please indicate your employment status following the restrictions introduced by the Italian Government since March, 9th 2020 ***

|__| Continued going to work at my workplace

|__| Working from home

|__| I had to stop working due to the emergency

|__| I am not employed

1. **Since March, 9th 2020, how many times do you go out during a week? ***

|__| Never

|__| 1

|__| 2-3

|__| 4-5

|__| 6 or more

1. **Since March, 9th 2020, do you use public transport go to work or to provide supplies***

|__| No, never

|__| Yes, 1-3 times per week

|__| Yes, 4-6 times per week

|__| Yes, 7 or more times per week

**Any additional information and comments**

__________________________________________________________________________________________________________________________________________________________________________________________________________________________________________

**Phase II questionnaire**

**Laboratory test results**

Please answer the questions below regarding the results of the laboratory tests you have carried out

**A1. Have you made one or more swabs with a POSITIVE result? ***

|__| Yes |__| No

**A2. Indicate the date of the FIRST POSITIVE Swab**

**_____/_____/_____**

**A3. With reference to your FIRST POSITIVE SWAB, enter the viral load, if available**

**_______________________________________________**

**A4. Indicate the number of elapsed days from the beginning of the symptoms to the day of the FIRST POSITIVE SWAB**

**__________________________________________________**

**A5. Have you made one or more swabs with a NEGATIVE result? ***

|__| Yes |__| No

**A6. Indicate the date of the LAST NEGATIVE SWAB**

**_____/_____/_____**

**A7. Have you performed the serological test too? ***

|__| Yes |__| No

**A8. Indicate the outcome of the serological test for Immunoglobulin G (IgG) Antibodies:**

|__| Positive

|__| Negative

**A9. Indicate the outcome of the serological test for Immunoglobulin M (IgM) Antibodies:**

|__| Positive

|__| Negative

**A10. Indicate the number of elapsed days from the beginning of the symptoms to the day in which the serological test was performed:**

**_________________________________________________**

**A11. Any additional information and comments**

______________________________________________________________________________________________________________________________________________________________________________________________________________________________________

**Thank you for your cooperation!**

**APPENDIX**

**Respondents and non-respondents to the Phase I and Phase II survey**

|  | **Respondents Phase I and Phase II** | **Respondents only to Phase I** |  |
| --- | --- | --- | --- |
|  | **(n° 2703)** | **(n° 4161)** | **p value** |
| **Age** | 49.6, ±14.7 | 46.2, ±13.1 | 0.014 |
| **Positive Nasopharyngeal swab** | 706 (26.1%) | 862 (20.7%) | <0.0001 |
| **Female** | 1841 (68.14%) | 2669 (64.1%) | 0.001 |
| **Ethnicity** | 2666 (98.60%) | 4133 (99.3%) | 0.005 |
| **Lower educational status** | 151 (5.6%) | 17 (0.4%) | <0.0001 |
| **Medium educational status** | 837 (31.0%) | 1019 (24.5%) |  |
| **High educational status** | 1715 (63.4%) | 3125 (75.1%) |  |
| **Self-reported comorbidities** |  |  |  |
| **Lung diseases (e.g. asthma, obstructive pulmonary disease)** | 196 (7.3%) | 297 (7.1%) | 0.859 |
| **Heart diseases (e.g. ischemic heart disease, atrial fibrillation)** | 103 (3.8%) | 120 (2.9%) | 0.034 |
| **Hypertension** | 361 (13.4%) | 567 (13.6%) | 0.748 |
| **Kidney diseases** | 22 (0.8%) | 33 (0.8%) | 0.925 |
| **Immune system diseases (e.g. thyroid disease, psoriasis, rheumatoid arthritis)** | 266 (9.8%) | 365 (8.8%) | 0.134 |
| **Tumours** | 88 (3.3%) | 121 (2.9%) | 0.413 |
| **Metabolic diseases (e.g. diabetes, obesity, gout)** | 124 (4.6%) | 161 (3.9%) | 0.145 |
| **Liver diseases (e.g. hepatitis, cirrhosis, liver failure)** | 21 (0.8%) | 29 (0.7%) | 0.703 |
| **Depression and/or anxiety** | 194 (7.2%) | 265 (6.4%) | 0.190 |

**APPENDIX**

Supplementary appendix

This appendix formed part of the original submission

(1) **Identification of Critical Factors by Exploratory and confirmatory factor analysis**

The factor structure of the COVID-19 self-reported symptoms items was tested using two procedures: Exploratory factor analysis (EFA) [1] and confirmatory factor analysis (CFA) [2]. Exploratory factor analysis (EFA) is a variable reduction technique which identifies the number of latent constructs and the underlying factor structure of a set of variables. Confirmatory factor analysis (CFA) is a statistical technique used to verify the factor structure of a set of observed variables. CFA allows testing the hypothesis that a relationship between the observed variables and their underlying latent constructs exists. In the first step, EFA and PA (parallel analysis) were performed to identify item performance (loadings) and to define the number of factors underlying the scale. EFA was applied using tetrachoric correlation matrix and two solutions were tested with one and two factors. PA was performed (1000 random correlation matrices) to establish the number of factors underlying the scale. Classical criteria from EFA were used: eigenvalue rule according Kaiser’s rule of thumb [3], only a factor with eigenvalue > 1 explained more of the variance, scree plot (n° of factors before the break in the eigenvalue plot), Horn’s parallel analysis (number of factors with eigenvalue higher than average random eigenvalues), and factor-loading rule (item-factor correlations >0.35).

**(2)** **Confirmation of presence of latent variable** **by** **Confirmatory Factor Analysis (CFA)**

A Confirmatory Factor Analysis (CFA) via Structural Equation Modeling (SEM) was performed to confirm the presence of a one latent variable (factors) underlying the COVID-19 self-reported symptoms items. The items (endogenous variables) are (S1) *Fever, with temperatures above 37*.*5 ° C for at least three consecutive days*, (S2) Cough, (S3) Sore throat and/or cold, (S4) Headache, (S5) Pain in muscles, bones, joints, (S6) Loss of taste and / or smell, (S7) Respiratory difficulty (sense of breathlessness at rest), (S8) Chest pain (sternum pain), (S9) Tachycardia, (S10) Gastrointestinal complaints (diarrhea, nausea, vomiting), (S11) Conjunctivitis (red eyes). Several goodness-of-fit criteria was use [4]: (i) Standardized Root Mean Square Residual (SRMR); (ii) Root Mean Square Error of Approximation (RMSEA). In general, a model is considered to show good fit when the SRMR and RMSEA are not higher than 0.10. In addition, further CFA indexes as gamma for unidimensionality (AVE; average variance extracted acceptable if > 0.4) and the general validity coefficient (McDonald's Omega acceptable if > 0.8) verify the adequacy of single-factor measurement model and the factor validity index (rho) for the evaluation of the correlation factor with the variables [5]. Finally, the standardized factor loadings (item-factor correlations) (acceptable if > 0.4) proved high-quality specific factor validity for each item and determinacy coefficient (DC) >0.90 as construct validity index.

**(3)** **Development of optimal scoring algorithm** **by Homogeneity Analysis**

After evaluating the unidimensionality through exploratory and confirmatory factor analyses an optimal questionnaire score by Homogeneity Analysis (Multiple Correspondence Analysis) [6] was constructed. Through the HOMALS procedure we replaced item dichotomous responses (Yes/No) with category quantifications: the score is the sum of the subject’s item responses, once they are recoded by category quantifications. Category quantifications assigned by HOMALS to the dichotomous items are named “optimal” quantifications because they maximize homogeneity among items and minimize measurement error. Subsequently, the COVID-19 self-reported optimal score was rescaled to a range of 0 -10 using: 10*( EPICOVID 19 score - min)/(max - min). The result of this equation was used as the EPICOVID 19 self-reported optimal score for each individual, a continuous trait that summarizes the common components of COVID-19 self-reported symptoms items. With more details the subject score is:


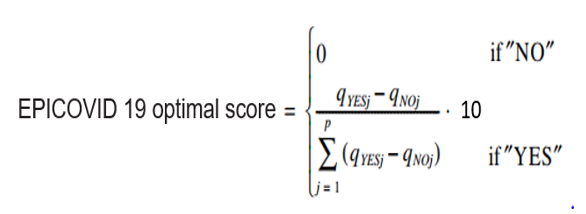


the figures qYESj and qNOj are the HOMALS optimal quantifications of the “Yes” and “No” binary item responses, respectively [6]. Additionally, Cronbach Index (α) which is a scale reliability coefficient based on the internal consistency, and Greenacre Index (τ) which is the percentage of inter-item association explained by the first dimension, were also computed

(4) **Validation of the Scoring algorithm by receiver operating characteristics (ROC)**

Hence, the score validation was performed by receiver operating characteristics (ROC) analysis was performed to evaluate the COVID-19 self-reported symptoms items effectiveness in distinguishing symptomatic individuals severity. With the aim of discriminating between different severity of symptoms, for each EPICOVID 19 factor score built, sensitivity, specificity, and youden’s index were computed with two reference standard [7]: a) subjects with NPS + vs subjects with NPS - ; b) subjects with serological IgG(+) vs subjects with IgG(-).Overall, predictive performance was measured by the AUC (Area Under the Curve): ROC curves with AUC of 0.5 indicating no predictive diagnosis versus AUC of 1.0 indicating perfect ones. The Youden index (J ), a statistic that captures the performance of a diagnostic test, computed as J = sensitivity + specificity - 1, was used to identify the optimal cut point [8]. All statistical analyses were done using R software (version 3.6.3)

1. Gorsuch RL. Common factor analysis versus component analysis: Some well and little known facts. *Multivariate Behavioral Research* 1990, **25**, 33-39. PMID: 26741966

2. Gifi A. Nonlinear multivariate analysis. New York: John Wiley & Sons 1990 (First edition 1981, Department of Data Theory, University of Leiden).

3. Bartholomew DJ, Knott M. Latent variable models and factor analysis. 1999, 2nd ed. New York: Oxford University Press.

4. Hooper D, Coughlan J, Mullen M. Structural Equation Modelling: Guidelines for Determining Model Fit. *Electronic Journal of Business Research Methods* 2008; **6**(1), 53-60.

5. McDonald RP. Theoretical foundations of principal factor analysis and alpha factor analysis. *Br J Math Stat Psychol* 1970; **23**:1-21.

6. Greenacre M. Correspondence analysis in the social sciences. London: Academic Press; 1994.

7. Choi BC. Slopes of a receiver operating characteristic curve and likelihood ratios for a diagnostic test. *Am J Epidemiol* 1998; **148**: 1127–32 . PMID: 9850136

8. Schisterman EF, Perkins NJ, Liu A, Bondell H. Optimal cut-point and its corresponding Youden Index to discriminate individuals using pooled blood samples. *Epidemiology* 2005;**16**:73e81 . PMID: 15613948

**APPENDIX**

**One factor solution extracted by Principal-Component Factors**

| Symptoms items | One-factor solution |
| --- | --- |
| (S1) Fever, with temperatures above 37.5 ° C for at least three consecutive days | 0.653 |
| (S2) Cough | 0.702 |
| (S3) Sore throat and/or cold | 0.571 |
| (S4) Headache | 0.721 |
| (S5) Pain in muscles, bones, joints | 0.815 |
| (S6) Loss of taste and / or smell | 0.732 |
| (S7) Respiratory difficulty (sense of breathlessness at rest) | 0.723 |
| (S8) Chest pain (sternum pain) | 0.736 |
| (S9) Tachycardia (Palpitation) | 0.707 |
| (S10) Gastrointestinal complaints (diarrhea, nausea, vomiting) | 0.762 |
| (S11) Conjunctivitis (red eyes) | 0.606 |

**APPENDIX**

**Standardized coefficients and goodness of fit indexes for one factor model.**

|  | Standardized coefficients | Std.Err | z-value | P(>\|z\|) |
| --- | --- | --- | --- | --- |
| (S1) Fever, with temperatures above 37.5 ° C for at least three consecutive days | 0.646 | 0.020 | 31.657 | <0.0001 |
| (S2) Cough | 0.666 | 0.020 | 33.816 | <0.0001 |
| (S3) Sore throat and/or cold | 0.537 | 0.023 | 23.752 | <0.0001 |
| (S4) Headache | 0.697 | 0.019 | 37.296 | <0.0001 |
| (S5) Pain in muscles, bones, joints | 0.814 | 0.016 | 51.722 | <0.0001 |
| (S6) Loss of taste and / or smell | 0.724 | 0.019 | 38.974 | <0.0001 |
| (S7) Respiratory difficulty (sense of breathlessness at rest), | 0.688 | 0.021 | 32.710 | <0.0001 |
| (S8) Chest pain (sternum pain) | 0.701 | 0.021 | 33.122 | <0.0001 |
| (S9) Tachycardia | 0.675 | 0.023 | 29.201 | <0.0001 |
| (S10) Gastrointestinal complaints (diarrhea, nausea, vomiting) | 0.737 | 0.018 | 42.009 | <0.0001 |
| (S11) Conjunctivitis (red eyes) | 0.557 | 0.027 | 20.373 | <0.0001 |
|  | AVE; average variance extracted | | | 0.516 |
|  | McDonald's Omega |  |  | 0.904 |
|  | Factor validity index (rho) | |  | 0.951 |
|  | SRMR-Standardized Root Mean Square Residual | | | 0.072 |
|  | RMSEA-Root Mean Square Error of Approximation | | | 0.052 |
|  | CFI-Comparative fit index | |  | 0.977 |
|  | TLI-Tucker-Lewis index |  |  | 0.971 |

**Sensitivity and specificity of EPICOVID19 DC compared to COVID-19 positive molecular and serological diagnosis (<60 years)**

|  | Tested for SARS-CoV-2 | | | |
| --- | --- | --- | --- | --- |
| <60 years | NASOPHARYNGEAL SWAB (2114) | | ANTIBODIES IgG  (399) | |
|  | (NPS+ 449 ) | | (IgG+  83 ) | |
| Statistic | Value | 95% CI | Value | 95% CI |
| Sensitivity | 80.85% | 76.90% to 84.38% | 81.93% | 71.95% to 89.52% |
| Specificity | 66.79% | 64.47% to 69.05% | 79.75% | 74.89% to 84.04% |
| Positive Likelihood Ratio | 2.43 | 2.24 to 2.64 | 4.05 | 3.18 to 5.15 |
| Negative Likelihood Ratio | 0.29 | 0.24 to 0.35 | 0.23 | 0.14 to 0.36 |
| COVID-19 Disease % | 21.24% | 19.51% to 23.05% | 20.80% | 16.92% to 25.12% |
| PPV | 39.63% | 37.69% to 41.60% | 51.52% | 45.50% to 57.48% |
| NPV | 92.82% | 91.42% to 94.01% | 94.38% | 91.37% to 96.38% |
| Accuracy | 69.77% | 67.77% to 71.73% | 80.20% | 75.95% to 84.00% |
| Cut-off | 2.57 | | 2.71 | |

**Sensitivity and specificity of EPICOVID19 DC compared to COVID-19 positive molecular and serological diagnosis (>=60 years)**

|  | Tested for SARS-CoV-2 | | | |
| --- | --- | --- | --- | --- |
| >=60 years | NASOPHARYNGEAL SWAB (589) | | ANTIBODIES IgG  (73) | |
|  | (NPS+ 245 ) | | (IgG+  25 ) | |
| Statistic | Value | 95% CI | Value | 95% CI |
| Sensitivity | 53.88% | 47.42% to 60.24% | 88.00% | 68.78% to 97.45% |
| Specificity | 64.83% | 59.52% to 69.87% | 89.58% | 77.34% to 96.53% |
| Positive Likelihood Ratio | 1.53 | 1.27 to 1.84 | 8.45 | 3.64 to 19.61 |
| Negative Likelihood Ratio | 0.71 | 0.61 to 0.83 | 0.13 | 0.05 to 0.39 |
| COVID-19 Disease % | 41.60% | 37.58% to 45.70% | 34.25% | 23.53% to 46.28% |
| PPV | 52.17% | 47.57% to 56.74% | 81.48% | 65.46% to 91.08% |
| NPV | 66.37% | 62.80% to 69.76% | 93.48% | 83.16% to 97.65% |
| Accuracy | 60.27% | 56.19% to 64.25% | 89.04% | 79.54% to 95.15% |
| Cut-off | 1.61 | | 1.28 | |
